# Optimisation of the air fraction correction for lung PET/CT: addressing resolution mismatch

**DOI:** 10.1186/s40658-023-00595-y

**Published:** 2023-12-05

**Authors:** Francesca Leek, Cameron Anderson, Andrew P. Robinson, Robert M. Moss, Joanna C. Porter, Helen S. Garthwaite, Ashley M. Groves, Brian F. Hutton, Kris Thielemans

**Affiliations:** 1 Institute of Nuclear Medicine, University College London Hospitals NHS Trust, London, UK; 2 Nuclear Medicine Metrology, National Physical Laboratory, Teddington, UK; 3 Christie Medical Physics and Engineering, The Christie NHS Foundation Trust, Manchester, UK; 4 Schuster Laboratory, School of Physics and Astronomy, University of Manchester, Manchester, UK; 5 Department of Medical Physics and Biomedical Engineering, University College London, London, UK; 6 UCL Respiratory, University College London and Interstitial Lung Disease Service, University College London Hospitals NHS Trust, London, UK; 7 Centre for Medical Image Computing, University College London, London, UK

**Keywords:** PET/CT, Air fraction correction, Quantification, Perturbation, Lung imaging, Resolution

## Abstract

**Background:**

Increased pulmonary $^{18}{}$F-FDG metabolism in patients with idiopathic pulmonary fibrosis, and other forms of diffuse parenchymal lung disease, can predict measurements of health and lung physiology. To improve PET quantification, voxel-wise air fractions (AF) determined from CT can be used to correct for variable air content in lung PET/CT. However, resolution mismatches between PET and CT can cause artefacts in the AF-corrected image.

**Methods:**

Three methodologies for determining the optimal kernel to smooth the CT are compared with noiseless simulations and non-TOF MLEM reconstructions of a patient-realistic digital phantom: (i) the point source insertion-and-subtraction method, $h_{pts}$; (ii) AF-correcting with varyingly smoothed CT to achieve the lowest RMSE with respect to the ground truth (GT) AF-corrected volume of interest (VOI), $h_{AFC}$; iii) smoothing the GT image to match the reconstruction within the VOI, $h_{PVC}$. The methods were evaluated both using VOI-specific kernels, and a single global kernel optimised for the six VOIs combined. Furthermore, $h_{PVC}$ was implemented on thorax phantom data measured on two clinical PET/CT scanners with various reconstruction protocols.

**Results:**

The simulations demonstrated that at $<200$ iterations (200 i), the kernel width was dependent on iteration number and VOI position in the lung. The $h_{pts}$ method estimated a lower, more uniform, kernel width in all parts of the lung investigated. However, all three methods resulted in approximately equivalent AF-corrected VOI RMSEs (<10%) at $\ge$200i. The insensitivity of AF-corrected quantification to kernel width suggests that a single global kernel could be used. For all three methodologies, the computed global kernel resulted in an AF-corrected lung RMSE <10%  at $\ge$200i, while larger lung RMSEs were observed for the VOI–specific kernels. The global kernel approach was then employed with the $h_{PVC}$ method on measured data. The optimally smoothed GT emission matched the reconstructed image well, both within the VOI and the lung background. VOI RMSE was <10%, pre-AFC, for all reconstructions investigated.

**Conclusions:**

Simulations for non-TOF PET indicated that around 200i were needed to approach image resolution stability in the lung. In addition, at this iteration number, a single global kernel, determined from several VOIs, for AFC, performed well over the whole lung. The $h_{PVC}$ method has the potential to be used to determine the kernel for AFC from scans of phantoms on clinical scanners.

## Background

There has been an increased interest in studying lung diseases, such as idiopathic pulmonary fibrosis (IPF), chronic obstructive pulmonary disease (COPD) and coronavirus, with positron emission tomography/computed tomography ( PET/CT). Several studies have shown that pulmonary uptake of fluorodeoxyglucose ($^{18}{}$F-FDG) is increased in these diseases, when compared to control subjects [1–12], and predicts measurements of health and lung physiology [3–5].

To better characterise the role of lung cells in promoting disease activity and progression, methods to measure lung cell metabolism specifically must be implemented, as highlighted in a position paper by Chen et al. [13]. Quantification is limited by several difficulties: the lung density and local volume changes during respiration, the anatomical mismatch between PET and CT, and the relative contributions of tissue, air and blood to the PET signal, known as the tissue fraction effect (TFE). Methods to correct for the density and volume changes during the respiratory cycle have been proposed [6, 14, 15].

The TFE is the result of the finite size of an imaging voxel, being significantly larger than the average alveolus size, leading to a single voxel containing multiple tissues and air. Correcting for this has been shown to alter image interpretation in patients with IPF [2, 6, 8]. The air fraction correction (AFC) adopts a simplified model of the lung where the observed activity concentration is assumed to be the radiotracer distributed throughout the cellular component of the tissue (parenchyma and blood), and a gas component, containing no activity [2]. The CT acquired for attenuation correction (AC) of PET data is utilised to determine voxel-wise fractions of aerated tissue. These air fractions (AF) can be used to account for the variable air content, thus providing an estimate of tracer uptake per gram of tissue.

The CT image is used for both AC and AFC; however, the difference in resolution between PET and CT can cause artefacts in the AF-corrected PET image. Preliminary simulation studies demonstrated that, when lung tissue uptake is uniform, resulting in a homogeneous AF-corrected images, the attenuation image used for AC should have the same resolution as the intrinsic PET scanner resolution, while the AF image should approximately have the reconstructed PET image resolution [16]. This study assumed respiratory phase matching between PET and CT, as will be done in this work.

Empirical measurements for characterising the reconstructed PET resolution employ point sources in air positioned at finely sampled locations throughout the field-of-view (FOV), reconstructed using filtered back projection (FBP), with no smoothing or apodisation [17]. This method does not, however, represent the scatter and noise conditions that are present when imaging a patient. It is therefore not a representative measure of image resolution for a patient scan, even when using FBP. This is even less the case for the statistical reconstruction methods used clinically. These methods are known to be nonlinear and object dependent, especially at low numbers of iterative updates; this contributes to a spatially variant image resolution [18]. Noise levels become unacceptable as iterations proceed for unregularised algorithms, such as maximum likelihood expectation maximisation (MLEM) and ordered subsets expectation maximisation (OSEM). To compensate, the algorithms are generally stopped after a predetermined number of iterations, potentially resulting in under-convergence in certain regions; post-reconstruction filters are employed to control any additional noise. Both will contribute to an altered image resolution.

The reconstructed image resolution is often estimated from the point spread function (PSF). A common method for determining the PSF in simulations is via the perturbation method [19], which adds projections of a small, noise-free, perturbation, e.g. a point source, to sinogram data. Subtracting the unperturbed reconstructed dataset from the perturbed, results in an estimation of the PSF, dependent on activity distribution, position in the FOV, and reconstruction settings. The non-negativity constraint of iterative algorithms can artificially enhance the apparent spatial resolution if the point source is reconstructed without any background activity [20]. It has been recommended that a non-zero background be added to the point source data before reconstruction to minimise these effects, hence, the point-source-insertion-and-subtraction method was developed [19]. Gong et al. conducted a detailed study on the extent of spatial blurring for hot spots, based on point source contrast and reconstruction algorithm [21]. However, to the best of our knowledge the extent of spatial blurring in (potentially under-converged) low count regions, as are commonly seen in diseased lung, has not yet been investigated.

As an alternative to the point source insertion-and-subtraction method, Joshi et al.  [22] determined the reconstructed resolution of clinical scanners from scans of the Hoffman brain phantom. A digital Hoffman phantom was smoothed in all three dimensions with incremental full-width-half-maximum (FHWM) Gaussian kernels to obtain a library of the digital phantom at various resolutions. The effective resolution was estimated by determining the smoothed digital phantom that was closest to the reconstructed image using a least squares approach. The advantage of this method over the point source insertion-and-subtraction method is that the former does not require simulation capabilities. It does, however, rely on an accurate digital representation of the measured object.

This paper investigates three different methodologies by which the image resolution, as a function of reconstruction algorithm and convergence, can be determined for smoothing of the CT for AFC. In simulation studies, the point source insertion-and-subtraction method was compared to two variations on the methodology proposed by Joshi et al. A single methodology was then selected and the feasibility of its application to measured phantom data was assessed.

## Methods

### Description of the AFC method

Hounsfield units (HUs) scale linearly to linear attenuation coefficients (LACs) in the lung [23, 24], thus the relationship between the fraction of tissue in each voxel, $V_t$, and lung LAC can be expressed as:

1 $$\begin{aligned} \mu _l = V_t\mu _t + (1-V_t)\mu _a \end{aligned}$$

where $\mu _l$, $\mu _t$, $\mu _a$ are the LACs for 511 keV photons in the mu-map lung voxel, tissue and air, respectively [2]. $\mu _a$ can be set to zero in good approximation. To obtain an AF-corrected PET image, the simulated attenuation (mu) map is smoothed with a 3D Gaussian kernel, down-sampled to PET voxel size, and the reconstructed emission image divided by $V_t$ on a voxel-wise basis.

### Simulations

Three different methods for determining the optimal kernel with which to smooth the CT for AFC were investigated with noiseless simulations and non-TOF reconstructions of a digital patient-realistic phantom.

**Fig. 1:**
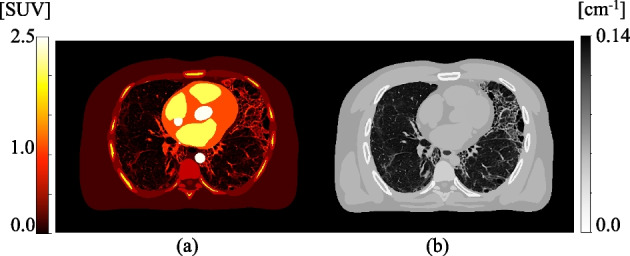
Modified digital XCAT phantom used for simulations (voxel size = $0.61 \times 0.61 \times 1.50 \hbox {mm}^3$); **a** ground truth emission (pre-AFC); **b** ground truth mu-map

**Fig. 2:**
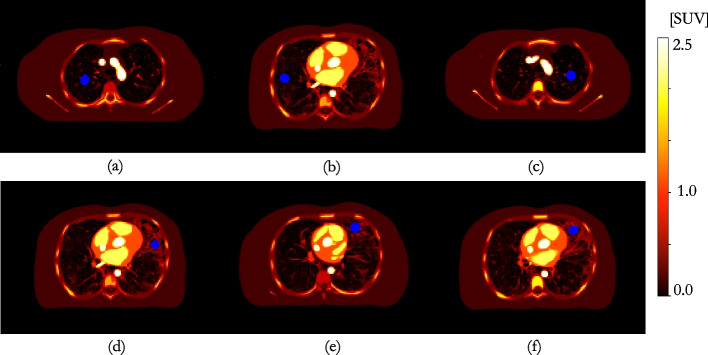
Axial slices through the modified XCAT depicting the six VOIs investigated in blue; top row shows VOIs in healthy lung and bottom row VOIs in fibrotic lung **a** $VOI1_{HL}$; **b** $VOI2_{HL}$; **c** $VOI3_{HL}$ **d** $VOI4_{IPF}$; **e** $VOI5_{IPF}$; **f** $VOI6_{IPF}$

**Fig. 3:**
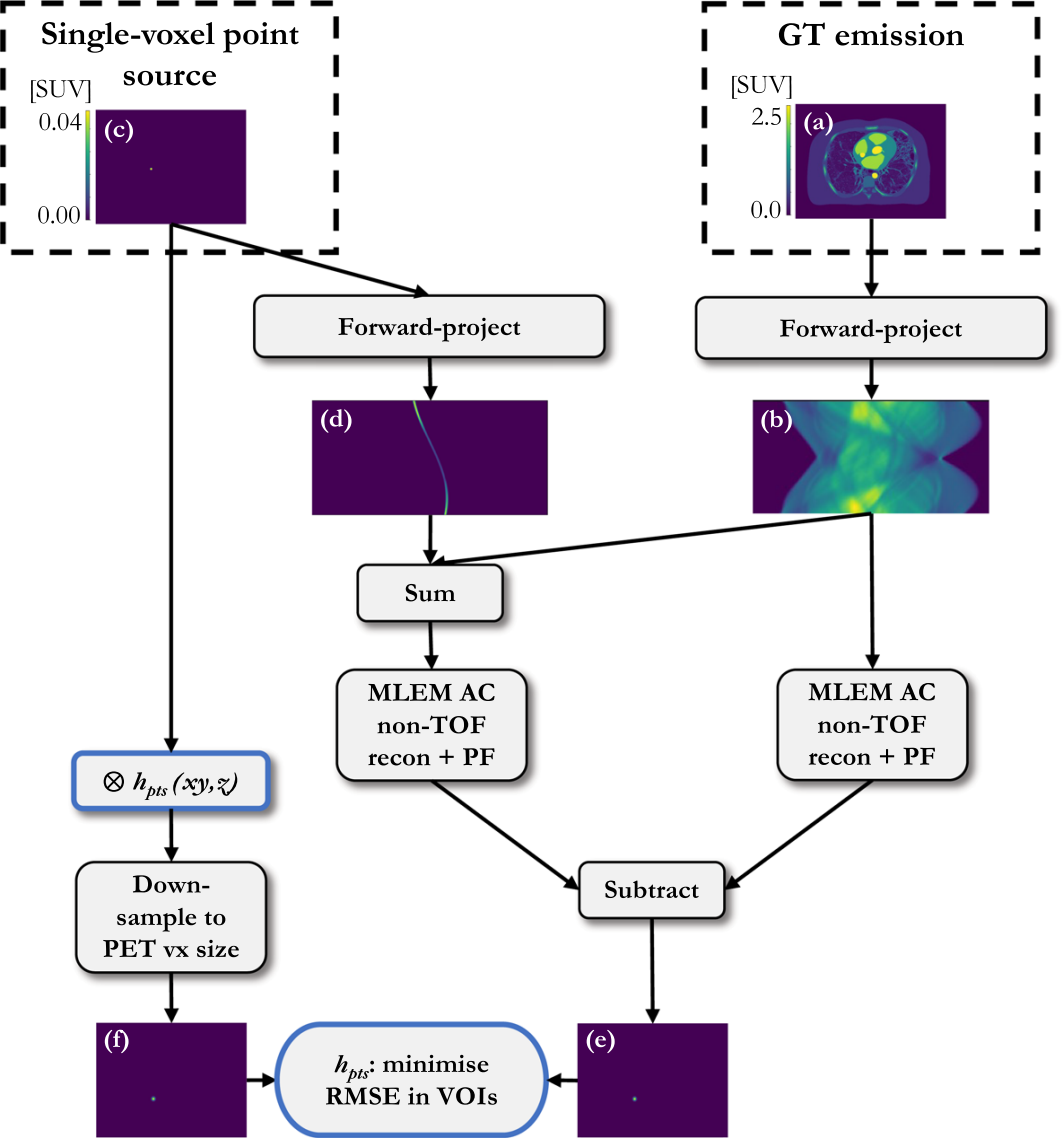
Workflow for the point source insertion-and-subtraction method for kernel determination; **a** simulated GT emission; **b** forward-projected and attenuated GT emission; **c** simulated single-voxel point source; **d** forward-projected and attenuated point source; **e** difference image between AC reconstructed and post-filtered ($h_{PF}$ = 6 mm) PET with perturbation and without; **f** simulated single-voxel point source convolved with $h_{pts}$ and down-sampled to PET voxel (vx) size. “Minimise RMSE in VOIs” refers to the six VOIs in the lung

A patient-realistic mu-map ground truth (GT) phantom was constructed by substituting the lungs in a digital 4D extended cardiac-torso (XCAT) phantom [25] with a diagnostic CT from an IPF patient, Fig. 1. This map was used to create an emission map of $^{18}{}F$ uptake. It was assumed that lung tissue uptake was uniform with a homogeneous AF-corrected SUV ($\mathrm {SUV_{AFC}}$) equal to 0.996, see phantom construction details in “Appendix 1”.

Data were simulated and reconstructed with a GE Discovery 710 (D710) PET template using STIR [26] via SIRF [27]. Both the emission and mu-maps were convolved with an isotropic Gaussian kernel with a 4.7 mm FWHM prior to forward-projection, to approximate the intrinsic resolution of the D710 [28]. All data were noiseless.

Non-time-of-flight (non-TOF) MLEM reconstruction, with 1000 iterations (1000 i), to investigate convergence, was performed into $2.71 \times 2.71 \times 3.27 \hbox {mm}^3$ voxels. MLEM, rather than OSEM, was chosen to avoid extra complications caused by the effect of subsets on convergence. The mu-map was smoothed with a kernel that matched the simulated intrinsic resolution of the PET scanner for AC [16]. A 6 mm FWHM post-reconstruction filter (PF) was applied to the reconstructed image, as may be done clinically.

**Fig. 4:**
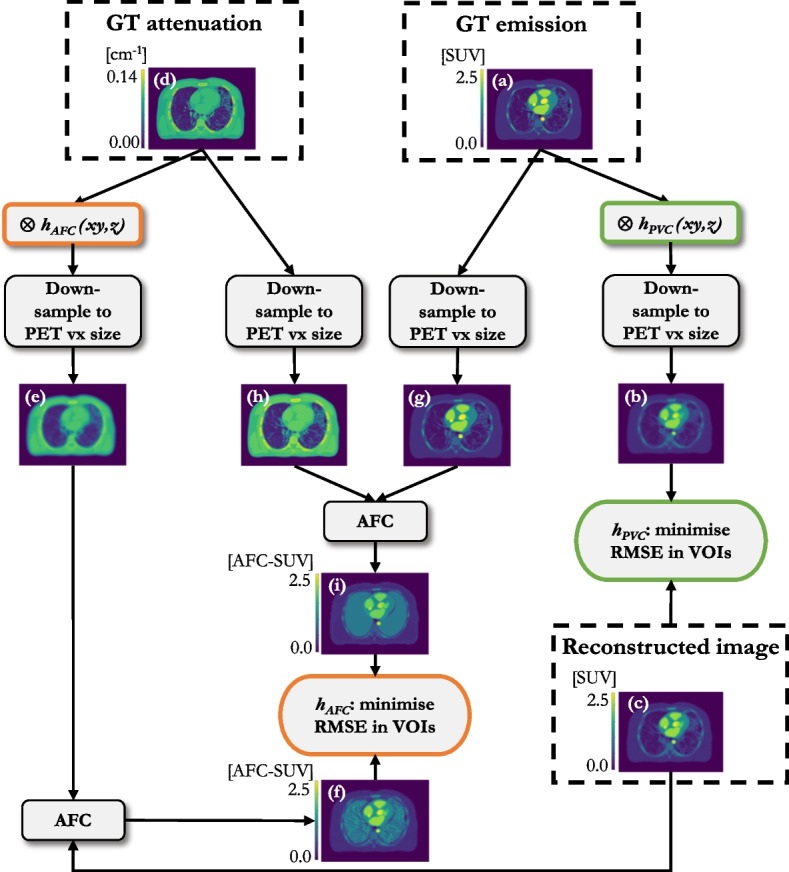
Workflow for the $h_{PVC}$ and $h_{AFC}$ methodologies for kernel determination; **a** simulated GT emission; **b** GT emission convolved with $h_{PVC}$ and down-sampled to PET voxel (vx) size; **c** AC non-TOF reconstructed and post-filtered ($h_{PF}$ = 6 mm) PET; **d** simulated GT mu-map; **e** mu-map convolved with $h_{AFC}$ and down-sampled to PET vx size; **f** AFC-AC PET (AC non-TOF reconstructed and post-filtered ($h_{PF}$ = 6 mm) PET divided by voxel-wise AFs, derived from (**e**)); **g** GT emission down-sampled to PET vx size; **h** GT mu-map down-sampled to PET vx size; **i** GT AF-corrected PET image. “Minimise RMSE in VOIs” refers to the six VOIs in the lung

The localised optimal kernel with which to smooth the mu-map for AFC was determined in six 20 mm diameter volumes of interest (VOIs)—three in healthy lung (HL) tissue, and three in regions of IPF (Fig. 2). A VOI diameter representative of that which might be quantitatively assessed clinically for interstitial lung disease was chosen; it was ensured the VOI was large enough (approximately twice the expected FWHM of the kernel) to reduce uncertainty on the kernel estimation. Three methods were investigated (as depicted in Figs. 3 and 4):

1. $h_{pts}$: the point source insertion-and-subtraction method using a single voxel point-source in the centre of the VOI (reconstructed contrast < 0.1, as recommended by Gong et al.  [21]). The GT point source image was smoothed by kernels of varying width; the reported $h_{pts}$ represents the kernel that resulted in the lowest root-mean-square-error (RMSE) with respect to the difference image (reconstructed emission subtracted from the reconstructed emission plus point source) in the VOI.
2. $h_{PVC}$: the GT emission was smoothed by kernels of varying width. The reported $h_{PVC}$ represents the kernel that resulted in the lowest RMSE in the VOI with respect to the reconstructed emission image, similar to Joshi et al.  [22].
3. $h_{AFC}$: the mu-map was smoothed by kernels of varying width and the reconstructed image AF-corrected with the smoothed mu-map; the reported $h_{AFC}$ denotes the kernel that resulted in the lowest RMSE with respect to the GT AF-corrected VOI.

For each method, the GT was convolved with 3D Gaussian kernels of decoupled in-plane and axial resolutions, of increasing FWHM (5–15 mm, 0.1 mm increments), before down-sampling to PET voxel size. For conciseness, only mean transaxial-axial kernel widths ($\textrm{FWHM}(xyz)$) are reported in this paper. The kernels estimated by each method were used to smooth the mu-map for voxel-wise AFC of the reconstructed data and the RMSE of each of the AF-corrected VOIs, with respect to the GT, was assessed. In addition, a whole lung volume (segmented on the high-resolution CT (HRCT), down-sampled to PET voxel size, and eroded by two voxels isotropically to avoid edge effects) was determined to quantify overall AFC performance.

### PET/CT acquisitions

The feasibility of determining the kernel with which to smooth the CT for AFC from scans of a physical phantom on clinical scanners was assessed. Both the $h_{pts}$ and $h_{AFC}$ methodologies are challenging to implement practically, the former due to the need for an accurate simulation program, and the later due to the presence of inactive insert walls in physical phantoms. Only the $h_{PVC}$ method was considered for physical phantom acquisitions.

**Fig. 5:**
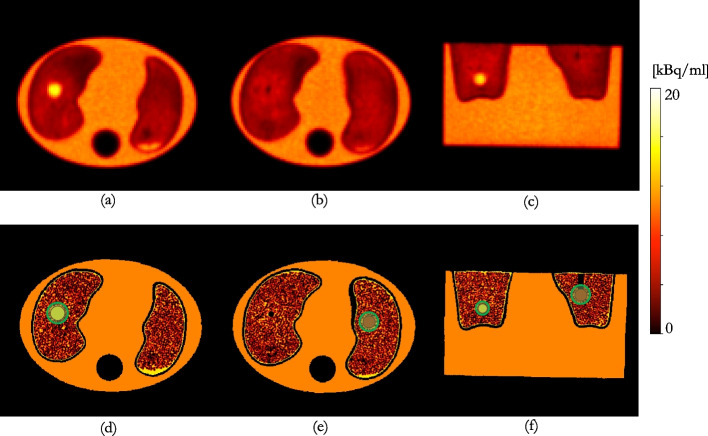
top row: Siemens “TOF” reconstruction; bottom row: GT constructed from HRCT of the phantom and known activity concentrations; **a**, **d** axial slice through the centre of the sphere in the right lung; **b**, **e** axial slice through centre of the sphere in the left lung; **c**, **f** coronal slice through the phantom showing both spheres. The VOIs positioned over the sphere inserts, combined into a single VOI, for kernel determination, are shown in green

**Fig. 6:**
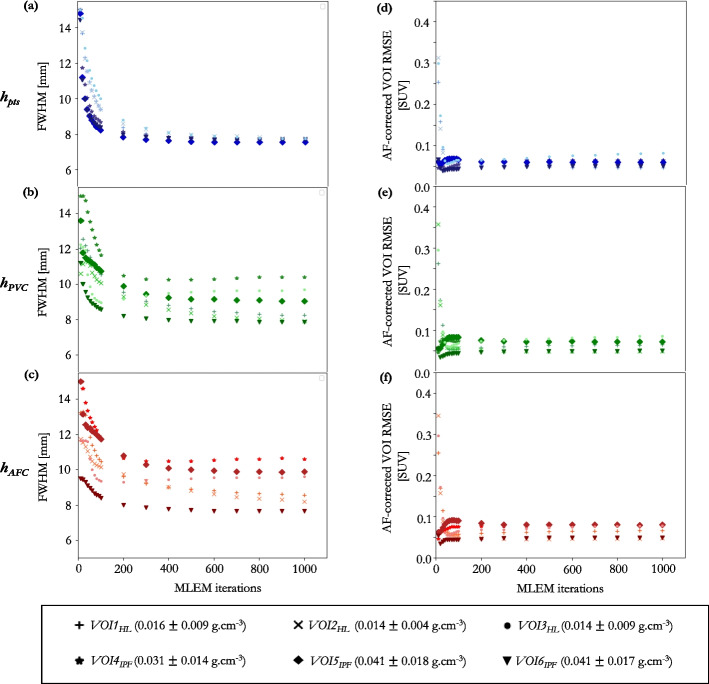
Simulated data: **a**–**c** estimated $FHWM_{xyz}$ for 10 to 1000 MLEM iterations for $h_{pts}$, $h_{PVC}$ and $h_{AFC}$ for each of the six VOIs, simulated on a GE D710 scanner in SIRF; **d**–**f** RMSE of the AF-corrected reconstructed VOIs at 10–1000 MLEM iterations; the mu-map was smoothed by the optimal kernel for each VOI determined for $h_{pts}$, $h_{PVC}$ and $h_{AFC}$, respectively. Each marker shape represents a VOI density (mean and 1$\sigma$)

The $h_{PVC}$ methodology was utilised to determine the optimal kernel for AFC on a D710 and a Siemens Biograph Vision 600 (Vision) at University College London Hospital (UCLH), using a commercially available elliptical thorax phantom (ECT/LUNGSPINE/I) [29]. The phantom contains two lung inserts containing polystyrene beads; the phantom was modified to allow the insertion of a 4 ml hollow sphere into the left lung and an 8 ml sphere into the right lung. The phantom was filled with $^{18}{}F$-FDG, with activity concentration ratios in the lung background and spheres such that the left lung was (approximately) homogeneous pre-AFC, and the right lung homogeneous post-AFC (sphere walls aside). A HRCT was acquired before the phantom was filled to provide a higher contrast between the Perspex walls and the fillable compartments, thereby allowing easier segmentation of the phantom components.

A GT emission was constructed from known activity concentrations, as measured in a radionuclide calibrator, and the segmented HRCT of the phantom (ITK-SNAP [30]), Fig. 5d–f. Further details on GT construction are given in “Appendix 2”.

**Table 1:** Reconstruction parameters for the clinical reconstructions assessed on the two clinical PET/CT scanners at UCLH. All PFs are Gaussian, except for the GE axial PF (a “standard” axial filter is a triangle filter with the ratio 1:4:1

Scanner	Reconstruction	Iterations	Subsets	Voxel size [$\hbox {mm}^3$]	PF FWHM [mm]
D710	VPFX (“EARL1”)	2	24	2.73 × 2.73 × 3.27	6.4 / “standard”
D710	VPFX	4	24	2.73 × 2.73 × 3.27	6.4 / “standard”
D710	QCFX	–	–	2.73 × 2.73 × 3.27	0.0
Vision	TOF	4	5	3.30 × 3.30 × 3.00	4.0
Vision	TOF+PSF	4	5	1.65 × 1.65 × 3.00	0.0
Vision	TOF (“EARL1”)	4	5	3.30 × 3.30 × 3.00	6.0

Clinical reconstructions from each scanner were assessed using the $h_{PVC}$ method, Table 1. GE’s “VPFX” (Vue Point FX) is a fully 3D TOF iterative reconstruction. This reconstruction is iterated to 2i clinically, and produces images in-line with “EARL1” standards [31]. A 4i reconstruction was also conducted to assess the effect of iterating for longer on the required smoothing for AFC. A 6.4 mm FWHM Gaussian PF in the transaxial plus a 1:4:1 ratio triangle filter in the axial was applied for both 2i and 4i reconstructions. GE’s “QCFX” reconstruction is a regularised iterative reconstruction algorithm, otherwise known as “Q.Clear”. Q.Clear is always run to convergence, therefore iterations and subsets are not specified inputs. TOF reconstructions, one that complies with “EARL1” (PF FWHM = 6.0 mm) standards [31], and one with a 4.0 mm FWHM PF were reconstructed on the Siemens scanner. All reconstructions were conducted using CT-based AC.

**Fig. 7:**
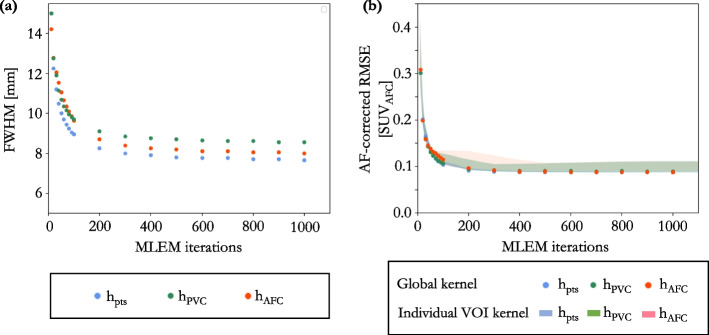
Simulated data: **a** estimated $\textrm{FWHM}_{xyz}$ for 10–1000 MLEM iterations for the global $h_{pts}$, $h_{PVC}$ and $h_{AFC}$ kernels determined from the six VOIs combined; **b** RMSE of the AF-corrected whole lung ($\rho$ = 0.024 ± 0.018 g.$\hbox {cm}^{-3}$) when the mu-map was smoothed by a global kernel derived from the six VOIs combined (dots), and kernels derived from individual VOIs (shaded regions depict range across six VOIs) at 10–1000 MLEM iterations

**Fig. 8:**
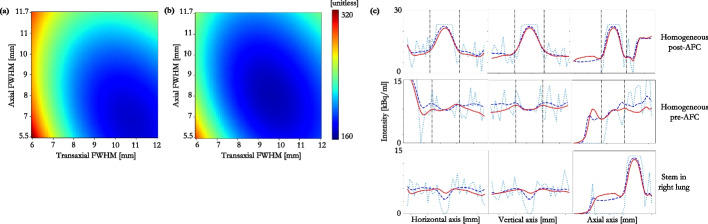
Measured data: **a** heatmap depicting nWMSE for each kernel combination investigated for GE D710 VPFX 2i reconstruction (minimum at $h_{PVC}$ = 10.7 × 10.7 × 7.2 mm$^3$); **b** heatmap depicting nWMSE for each kernel combination investigated for GE D710 VPFX 4i24s reconstruction (minimum at $h_{PVC} = 9.6 \times 9.6 \times 8. 5\,mm^3$); **c** horizontal, vertical and axial profiles through the homogeneous post-AFC sphere (top row), the homogeneous pre-AFC sphere (middle row) and a cold Perspex stem (bottom row). The down-sampled GT emission (light blue, dotted), VPFX 2i reconstructed image (red, solid), GT emission smoothed by the $h_{PVC}$ estimated kernel and down-sampled (royal blue, dashed) are depicted; the extent of the VOI used for kernel determination, in each orientation is shown by the black dash-dotted line

**Fig. 9:**
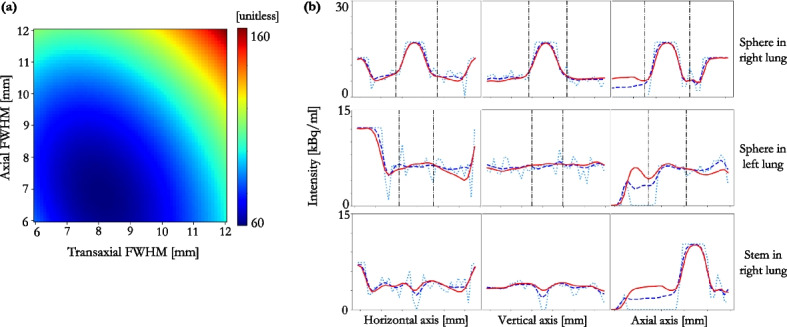
Measured data: **a** heatmap depicting nWMSE for each kernel combination investigated for Siemens Biograph Vision 600 TOF reconstruction (minimum at $8.1 \times 8.1 \times 6.8 {\rm mm}^3$); **b** horizontal, vertical and axial profiles through the sphere filled to be homogeneous post-AFC (top row), the sphere filled to be homogeneous pre-AFC (middle row) and a cold Perspex stem (bottom row). The down-sampled GT emission (light blue, dotted), TOF reconstructed image (red, solid), GT emission smoothed by the kernel and down-sampled (royal blue, dashed); the extent of the VOI in each orientation is shown by the black dash-dotted line

**Fig. 10:**
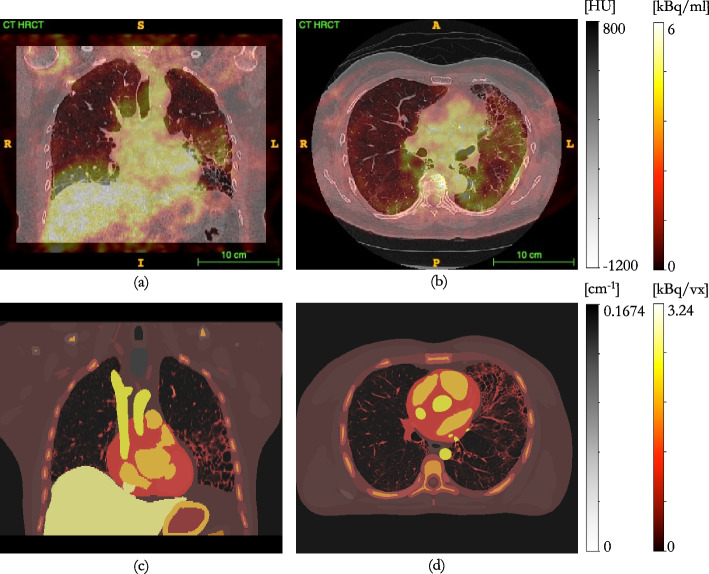
Fused PET/CT of patient with severe IPF, un-AF-corrected **a** coronal slice; **b** axial slice; fused attenuation and GT activity map patient realistic XCAT phantom **c** coronal slice; **d** axial slice

**Fig. 11:**
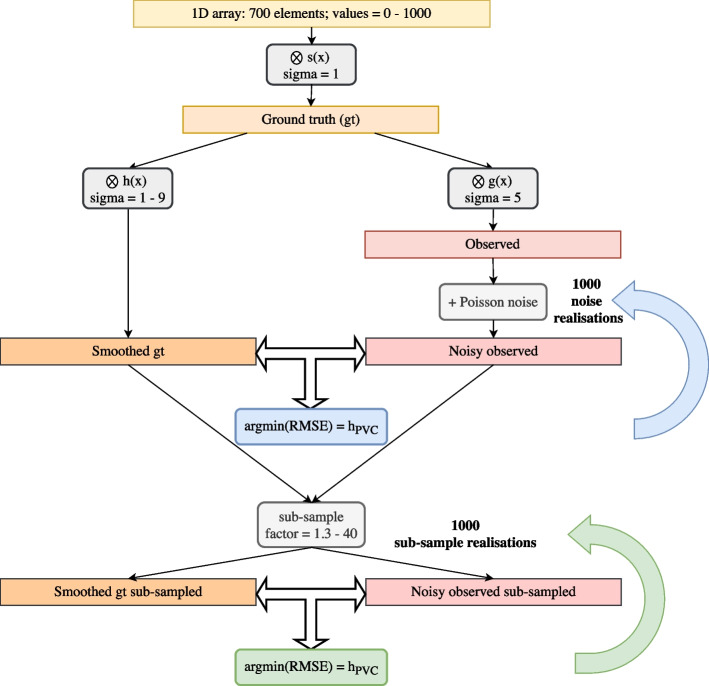
Workflow for uncertainty estimation on $h_{PVC}$ from 1000 sub-sampling realisations of a simulated 1D array

The optimal kernel for AFC was determined via the $h_{PVC}$ methodology. The spherical inserts were segmented on the HRCT, the mask was down-sampled to PET voxel size, and then dilated by two voxels isotropically, to ensure that artefacts caused by a mismatched kernel were analysed. While the simulations were noiseless, the acquired data were not. Therefore, a weighted-mean-squared-error (WMSE) of each of the activity concentrations in the smoothed GT activity VOI voxels ($A_{\textrm{true},v})$ to the activity concentrations in the reconstructed image VOI voxels ($A_{\textrm{obs},v}$) were calculated, Eq. 2. As a reasonable first approximation, it was assumed that the variance was proportional to the mean voxel value [32]. Each WMSE was normalised to the mean lesion value for each reconstruction (nWMSE), where *N* is the number of voxels in the VOI.

2 $$\begin{aligned} \textrm{nWMSE} = 1/N \sum _v{(A_{\textrm{obs},v} - A_{\textrm{true},v})^2 / A_{\textrm{obs},v}} \end{aligned}$$

The $h_{PVC}$ that resulted in the smallest nWMSE in the VOI, with respect to the reconstructed image, was reported.

The GT emission was smoothed by the estimated kernel for each reconstruction and the RMSE from the reconstructed image was assessed within the VOI dilated by an additional two voxels isotropically. This larger VOI was used for analysis to be representative of a VOI used to assess diffuse disease, e.g. IPF.

The uncertainty on the determined kernel FWHM was estimated by computing the standard deviation of $h_{PVC}$ FWHMs derived by randomly sampling half of the VOI voxels. Details on the voxel sub-sampling methodology can be found in “Appendix 3”.

## Results

### Simulations

The simulated data demonstrated that for fewer than 200i the kernel width was dependent on iteration number and VOI position in the lung, Fig. 6a–c. For 200i or more, each VOI had converged to a stable ($\Delta$ $\textrm{FWHM}(xyz)<$ 0.5 mm) estimate of kernel FWHM, independent of iteration number.

**Table 2:** Simulated data: mean estimated kernel $\textrm{FWHM}(xyz)$ of the six VOIs for each methodology at 200i or greater, and the resultant AF-corrected VOI RMSE when these kernels are applied to the CT for AFC

Method	Kernel FWHM(xyz) [mm]	AF-corrected VOI RMSE [%*SUV*_AFC_]
$h_{pts}$	7.82 ± 0.25	6.07 ± 1.25
$h_{PVC}$	9.00 ± 0.87	6.51 ± 1.25
$h_{AFC}$	9.23 ± 0.99	6.56 ± 1.37

In each case, the quoted uncertainty is one standard deviation across the six VOIs

A large inter-VOI variation in kernel width estimates was observed between methodologies (range = 7.6–14.1 mm). The $h_{pts}$ methodology estimated a lower, more uniform, $\textrm{FWHM}(xyz)$ across all six VOIs, see Fig. 6a–c. Even at 200i or more, a large inter-VOI kernel width variation was still observed, Table 2. However, at these higher iterations, all three kernel determination methods result in approximately equivalent AF-corrected RMSE in their respective VOIs (6.3 ± 1.3 $\%$ for all VOIs; range = 4.6–8.8 $\%$), Fig. 6d–f. This suggests that a single global kernel could be applied to AF-correct the whole lung. To assess this, the six VOIs were combined to determine a global kernel for each methodology. At 200i or more, the resultant FWHM ranged between 7.7 and 9.5 mm, depending on methodology, Fig. 7a. When this global kernel was applied in the voxel-wise AFC of the whole lung volume, the RMSE was consistent across all the methods at 200i or greater (range = 8.8–9.6$\%$), Fig. 7b. Using a kernel derived from a single VOI to AF-correct the whole lung resulted in a greater whole lung RMSE than when a global kernel was used, for all methodologies, as can be seen from the shaded areas in Fig. 7b. The RMSE of each AF-corrected individual VOI was less than 10$\%$, at as low as 30i, when the global kernel was used to AF-correct.

Reducing the VOI diameter increased the uncertainty on the estimated kernel width (individual VOI $h_{PVC}(xyz)$ = 8.6 ± 0.6 mm (mean ± 1$\sigma$) for 20 mm diameter VOI; $h_{PVC}(xyz)$ = 9.1 ± 1.0 mm for 4 mm diameter VOI).

For comparison, if a kernel is used that matched the resolution of the simulated PET scanner, even at 200 or more MLEM iterations, the minimum VOI RMSE is 13.1 $\%$ (for $\textrm{VOI2}_{HL}$ at 1000i).

### PET/CT acquisitions

Heatmaps of the nWMSE in the VOI for each $h_{PVC}$ pair are displayed for the clinical TOF reconstructions on each scanner in Figs. 8a and 9a. The nWMSE heatmap for the 4i 24 subsets (4i24s) reconstruction on the D710 is shown in Fig. 8b. The nWMSE was lower for the clinical Siemens TOF reconstruction than the clinical GE TOF reconstruction, Figs. 8a and 9a.

Profiles through each of the spheres, in all three dimensions, for the TOF reconstructed image, the unsmoothed GT, and the smoothed GT that was closest, in the least squares sense, to the reconstructed image, are shown in Figs. 8c and 9b.

The heatmaps show that the minimum nWMSE, and therefore the optimal kernel to smooth the CT for AFC, was ill-defined for all clinical reconstructions investigated. The transaxial-axial FWHM kernel pair that resulted in the nWMSE minimum for each reconstruction, are shown in Table 3. The greatest uncertainty was associated with the kernel width determination in the axial direction, as demonstrated by the asymmetry of the heatmap gradients; this was seen for all reconstructions, Table 3.

**Table 3:** Measured data: transaxial and axial $h_{PVC}$ FWHM that resulted in the smoothed GT emission image that best matched the reconstructed image

Scanner	Reconstruction	$h_{PVC}(xy)$ FWHM [mm]	$h_{PVC}(z)$ FWHM [mm]
D710	VPFX 2i (“EARL1”)	10.7 ± 0.06	7.2 ± 0.39
D710	VPFX 4i	9.6 ± 0.13	8.5 ± 0.25
D710	QCFX	8.9 ± 0.14	7.2 ± 0.18
Vision	TOF	8.1 ± 0.11	6.8 ± 0.15
Vision	TOF+PSF	6.7 ± 0.05	4.3 ± 0.18
Vision	TOF (“EARL1”)	9.3 ± 0.07	8.3 ± 0.64

Uncertainty is one standard deviation of 100 voxel sub-sampling realisations, see “Appendix 3”

The profiles through the spheres show that the GT emission, smoothed by the estimated $h_{PVC}$ kernel, matches the reconstructed image well, both within the VOI and in the lung background. As both spheres contained activity concentrations, equivalent to, or greater than, lung background, a profile was drawn through the inactive Perspex stem of the right sphere; this was to assess the applicability of the smoothed CT to AF-correct the reconstructed PET within a low count region, as may be found in regions of diseased lung. Figures 8c and 9b show the $h_{PVC}$ smoothed GT emission matches the reconstruction within 10 $\%$, even within these regions.

The mean transaxial-axial kernel width for “EARL1” reconstructions was consistent between scanners (D710 $h_{PVC}(xyz)$ FWHM = 9.40 ± 0.32 mm; Vision $h_{PVC}(xyz)$ FWHM = 8.97 ± 0.52 mm). Increasing the number of iterations from two to four for the “EARL1” reconstruction on the D710 did not significantly alter the mean transaxial-axial kernel width estimation (2i $h_{PVC}(xyz)$ FWHM = 9.40 ± 0.32 mm; 4i $h_{PVC}(xyz)$ FWHM = 9.10 ± 0.24 mm).

The narrowest kernel width was obtained for the standard “TOF+PET” reconstruction on the Siemens Vision, which has the highest resolution of those investigated. This reconstruction also had the highest AF-corrected RMSE ($\%$ RMSE = 9.2), Table 4. The lowest was for the “EARL1” reconstruction on the Vision (RMSE = 5.5 $\%$), which employs the largest PF of those investigated. VOI RMSE was below 10$\%$ for all reconstructions (range = 5.5–9.2 $\%$).

## Discussion

The simulation study demonstrated that the FWHM estimates stabilised after approximately 200 non-TOF MLEM iterations, Figs. 6a–c and 7a.

The three different kernel determination methods estimate different kernel widths for CT smoothing for AFC, even on images from a large number of MLEM iterations, Figs. 6a–c and 7a. Despite this, an AF-corrected RMSE of less than 10 $\%$ was achieved for all three methods at 200 MLEM iterations or more, Figs. 6d–f and 7b. This suggests that, for this test data, AF-corrected quantification is not very sensitive to the smoothing applied to the CT for AFC. This could be due to the RMSE measures being insensitive to kernel widths in VOIs with little contrast, implying that determining $h_{PVC}$ (*resp.* $h_{AFC}$) on VOIs within which there is a relatively uniform activity concentration before (*resp.* after) AFC is numerically unstable. Note that $h_{pts}$ always fits on data with contrast by design of the method. Reducing the VOI diameter increased the uncertainty on the estimated kernel width, as would be expected with the corresponding decrease in contrast within the VOI.

The RMSEs in the AF-corrected lung were comparable (RMSE range = 8.8–9.7 $\%$ at greater than 200i), regardless of the kernel determination method. The simulation results in Fig. 6b provide confidence in the single global kernel approach, as the RMSE in the AF-corrected whole lung was lower with respect to using any of the kernels derived from an individual VOI. As noted above, the kernel width estimation derived from a single region is unstable, this instability is reduced by combining the VOIs. When the global kernel is used to AF-correct the individual VOIs, the RMSE was comparable to using the localised kernel for that VOI (global kernel RMSE range = 4.2–8.5$\%$; localised kernel RMSE range = 4.6–8.8$\%$, at 200i or more).

The $h_{PVC}$ method is the most practical to implement on measured data as $h_{pts}$ requires accurate simulation capability and $h_{AFC}$ needs VOIs that are uniform after AFC, which is difficult to achieve in phantom studies. It was therefore solely the $h_{PVC}$ method that was implemented on physical phantoms.

**Table 4:** Measured data: RMSE in the reconstructed VOI with respect to the GT emission convolved with $h_{PVC}$

Scanner	Reconstruction	GT VOI mean [kBq/ml]	VOI RMSE [kBq/ml]	VOI RMSE [$\%$]
D710	VPFX 2i (“EARL1”)	10.1 ± 2.5	0.847 ± 0.009	8.4 ± 0.04
D710	VPFX 4i	10.1 ± 2.5	0.829 ± 0.003	8.2 ± 0.01
D710	QCFX	10.1 ± 2.6	0.907 ± 0.004	9.1 ± 0.02
Vision	TOF	6.9 ± 2.1	0.435 ± 0.007	6.4 ± 0.11
Vision	TOF+PSF	7.3 ± 2.9	0.667 ± 0.003	9.2 ± 0.02
Vision	TOF (“EARL1”)	6.9 ± 2.0	0.385 ± 0.003	5.5 ± 0.05

Uncertainty on RMSE is one standard deviation of 100 voxel sub-sampling realisations, see “Appendix 3”. Uncertainty on GT VOI mean is one standard deviation over all voxels in the original VOI

**Table 5:** XCAT parameters

Parameter	Setting
Respiratory motion	Full inhale
Cardiac motion	None-end-diastole
Arm position	Not in the field-of-view
Voxel size (mm)	$0.607 \times 0.607 \times 1.5$

Figure 9a shows that the nWMSE was lower for the clinical Siemens TOF reconstruction than the clinical GE TOF reconstruction, Fig. 8a. This could be due to the slightly larger reconstructed voxel size used by Siemens, resulting in higher counts per voxel, and therefore less uncertainty on the reconstructed images (Table 4).

The RMSE in the reconstructed VOI for the Siemens TOF+PSF reconstruction was the highest of all reconstructions, potentially due to the edge artefacts commonly observed with PSF-based image reconstruction or the increased average variance in the smaller voxels. The smaller voxel size also contributes to the lowest $h_{PVC}(xyz)$ kernel FWHM.

A faster convergence of the estimated kernel widths for the simulations would be expected with TOF reconstruction [33]. In the lung in particular, Emond et al.  showed that 1600 non-TOF MLEM iterations were needed for the mean difference between the last two iterations to be less than 0.1 $\%$ overall, while only 240 MLEM iterations were needed for a 550 ps TOF FWHM system [34]. For the results of this paper, 200 non-TOF MLEM iterations took 01:00:12 (hh:mm:ss) on an Apple MacBook Pro (2.4 GHz Quad-Core Intel Core i5; memory: 16 GB); a recent implementation of STIR allows GPU enabled reconstruction via the parallelproj library [35], which resulted in a 12-fold reduction in this reconstruction time on a AMD Ryzen 9 5900 12-Core Processor with GEForce RTX 3070 GPU. The effect of OSEM and TOF reconstruction convergence on the CT smoothing required for AFC will be investigated in future work.

**Table 6:** 3D ANTs registration parameters

Parameter	Setting
Transformation model	Symmetric diffeomorphic (SyN)
Transformation	fast symmetric normalisation: affine + deformable transformation, with mutual information as optimisation metric. Gradient-descent step-size: 3
Similarity metric	Fast cross-correlation (window radius:4; weight: 1)
Optimisation	4 level resolution pyramid (25, 50, 25, 5 iterations)
Regularisation	12$\sigma$ FWHM Gaussian on the gradient; 3$\sigma$ FWHM on the deformation field

It has been suggested that a major factor contributing to uncertainty in quantification is the uncertainty in the delineation of the VOI [36]. In this paper, the VOI was drawn on the CT data set and copied to the registered PET image. The coordinates of the original boundary will therefore be rounded to the nearest voxel coordinates of the PET image, adding to uncertainty. The effect of VOI size was investigated for the GE VPFX 2i (“EARL1”) reconstruction, by dilating the down-sampled spherical mask by 0, 1, 2, 3 and 4 voxels isotropically. The range of $h_{PVC}(xyz)$ FWHMs for varying VOI diameters was 8.75–9.55 mm, resulting in RMSE in the reconstructed VOI in the range 8.22–8.27 $\%$. These results suggest that, for this experimental design, the AFC kernel was relatively insensitive to VOI size, provided that the edges of the lung were not included in the VOI.

The weighting strategy on mean-square-error (MSE) employed in this paper for determining the kernel for measured data was based on noise. Looking at the impact of balancing noise and application specific metrics could be investigated.

The optimal kernel to smooth the CT for AFC was ill-defined for all clinical reconstructions. Non-spherical/non-symmetrical VOIs may aid in differentiation between the transaxial and axial kernels, and may potentially offer a more unique solution. Alternatively shaped inserts, which can be embedded within the lungs of the thorax phantom, are being investigated.

This methodology paper uses an HRCT to create a GT for measured data. Clinically, an HRCT of patient data might not be available, thus patient PET data might need to be AF-corrected with a smoothed low-dose CT acquired for AC (CTAC). To assess clinical translation of the methodology, the use of the CTAC for kernel determination, for application to patient data, will be investigated in future work.

The ability to determine scanner- and reconstruction-specific kernels with which to smooth the CT for the AFC of patient data, relies on phantoms that are easy to prepare and scan reproducibly, and approximate the characteristics of diseased lung. It is hoped that the results from this work contribute to the design of such a phantom.

## Conclusions

Simulations of an IPF patient-realistic digital phantom for a non-TOF PET scanner indicated that a large number of MLEM iterations (around 200) were needed to approach image resolution stability. AF-corrected quantification was shown to not be very sensitive to the smoothing applied to the CT, indicating that a single global kernel could be applied to the CT for determination of AFs, with which to AF-correct the whole lung, on a voxel-wise basis. Moreover, a kernel derived on combined VOIs has been shown to be more numerically stable. The most practical method to determine the kernel for AFC is to smooth a GT emission image to match the reconstructed image, $h_{PVC}$. It has the potential to be used to determine the kernel for AFC for a clinical scanner from scans of physical phantoms, not just for $^{18}{}$F-FDG, but for other PET tracers used to study the lung. However, the construction of the ground-truth for phantoms containing lung-like structures is non-trivial due to registration and segmentation accuracy. This method is not limited to the determination of the AFC kernel but could also be utilised to determine the PSF for partial volume correction for both lung disease and tumour imaging.

## Data Availability

The datasets generated and/or analysed during the current study are not publicly available due Institutional Review Board permissions but are available from the corresponding author on reasonable request.

## Appendix 1: digital patient-realistic phantom construction

An IPF patient-realistic digital phantom was created by modifying the 4D extended cardiac-torso (XCAT) phantom, Fig. 10. The near-homogeneous structure in the XCAT lungs were replaced by the lungs of a patient with severe IPF. This allowed the effect of the disease structure on measures of resolution to be assessed whilst keeping the structure outside of the lungs relatively uniform. The simplicity of the phantom outside of the lungs aims to reduce computation time should the phantom be used as input to Monte Carlo modelling at a later date. The resolution of the phantom was maximised i.e., matched to the patient HRCT resolution, to preserve data in the construction of the GT.

The phantom construction steps were as follows:

1. *XCAT phantom simulation:* The XCAT phantom software [25] was used to create a 3D voxelised phantom. The parameters used are listed in Table 5. The voxel size was matched to the patient HRCT data. The resultant attenuation map was cropped to the thorax, to match the patient HRCT axial coverage, and the LAC units were converted from voxel$^{-1}$ to cm$^{-1}$.
2. *Patient data acquisition:* A single patient with severe IPF was identified from a study to assess the role of coagulation in IPF, conducted at UCLH. The patient underwent a PET/CT acquisition on a GE Discovery 710 as part of a research study. An HRCT (voltage: 100 kVp, current: 149 mA, exposure time: 1.095 s, voxel size: $0.607 \times 0.607 \times 1.5$ mm, pitch: 0.52) was acquired at end-expiration, in addition to the PET and CTAC scans. The HUs were converted to LACs [23].
3. *Segmentation of XCAT and patient lungs for registration:* The patient lungs were segmented on the HRCT using the “active contour segmentation mode” with thresholding, in ITK-SNAP (v3.8.0) [30]. Each XCAT lung was semi-automatically segmented using the same methodology as was used for the patient lungs. Mislabeling of vasculature and airways was manually corrected for such that they were included in the segmented lung. All segmentations were saved as NIfTIs.
4. *Registration of patient lungs to XCAT lungs:* Both the XCAT and patient lung masks were binary dilated by a $7\times 7\times 1$ box kernel using fslmaths; this ensured lung edges were included to assist registration. Patient lungs were registered to the XCAT attenuation map with Advanced Normalisation Tools (ANTs v2.3.5) [37] using symmetric diffeomorphic (differentiable map with differentiable inverse) transformation with the parameters listed in Table 6. Registration was conducted only within the dilated lung masks and for each lung separately.
5. *Replacement of XCAT lungs with IPF patient lungs:* The XCAT lung values were replaced by the registered patient data lung values. In regions where the registration was not deemed acceptable, i.e. too much warping of the patient lung, which occurred at the very base of one lung and a few voxels at the apex of both lungs, a value representative of the surrounding lung voxels was homogeneously assigned within that region.
6. *Creation of GT activity map:* Each organ outside of the lungs was segmented by their near-homogeneous XCAT voxel values. Each organ voxel was replaced by an activity concentration representative of IPF patient uptake. In the analytic simulations conducted in this work, only the ratios, not the units, of activity distribution are relevant; for convenience values are referred to in units of $\textrm{SUV}$ throughout. The activity concentration in each lung voxel was weighted by the patient lung LACs, such that the AF-corrected lung was homogeneous. The value for AF-corrected lung uptake was derived as follows: normal physiologic skeletal muscle uptake varies between 0.5 and 2.2 $\mathrm {SUV_{max}}$ [38]; a representative SUV of 1.0 was assumed. Holman et al. [6] demonstrates an approximate $\mathrm {SUV_{AFC}}$ of 3 in the lungs, in this patient cohort. An AF-corrected uptake ratio of 3:1 between lungs and muscle was therefore assumed. The patient data used to construct the phantom had a mean uptake in muscle of 0.332, therefore, a homogeneous AF-corrected uptake, equal to 0.996, was set in the lungs.

## Appendix 2: methodology for creating a ground truth emission for kernel determination for measured phantom data

1. An HRCT of the empty (before filling with a solution of activity concentration) thorax phantom was acquired on a single scanner to enable clearer definition of Perspex walls of phantom; this higher contrast enables easier segmentation. Reconstruction with a sharp filter was used for clearer edge definition.
2. The phantom was filled with the solution of activity concentration, according to experimental design.
3. An HRCT, CTAC and a PET scan were acquired on all clinical scanners.
4. All PET and CT scans of the phantom were converted from DICOM to interfile using the stir_math functionality.
5. The “empty” phantom HRCT (floating) was registered to the “full” phantom HRCT (reference). An initial rigid registration was conducted using NiftyReg reg_aladin [39].
6. The scanner beds were removed from the original “full” phantom and the partially-registered “empty” phantom to ensure that the registration was constrained to phantom features only.
7. The partially-registered “empty” phantom (floating) was registered with the “full” phantom (reference), with both beds removed.
8. The phantom components were segmented on the registered “empty” phantom in ITK-SNAP (v3.8.0) [30]. Each segmentation was saved as a NIfTI.
9. All segmentations were imported into STIR [26] as NIfTIs.
10. The centre-of-mass (COM) of each sphere and stem was computed.
11. STIR objects were created for each of the spheres (value: $-1$), sphere walls (value: 1) and stems (value: 1), using the COMs and published/estimated values for dimensions. Each object was saved as a NIfTI.
12. STIR objects were imported into STIR as NIfTIs.
13. Sphere masks (-sphere), sphere wall masks (sphere wall + sphere) and stem masks (stem—sphere wall) were created. All were saved as NIfTIs.
14. The HRCT of the “full” phantom was converted from HUs to LACs using bilinear conversion detailed in Carney et al. [24] for a Siemens scanner and Burger et al. [23] for a GE scanner.
15. An activity fraction map was created within the lung background:
  3 $$\begin{aligned} \mu _v = V_p\mu _p + V_w(\mu _w-\mu _s) + \mu _s(1-V_p) \end{aligned}$$
  where $\mu _v$, $\mu _p$, $\mu _w$ and $\mu _s$ are the LACs for the voxel, Perspex, water and polystyrene respectively, and $V_p$ and $V_w$ are the fractional volume of Perspex and water respectively. In this case, $\mu _w$ and $\mu _s$ were 0.0960 cm${}^{-1}$ and 0.0079 cm${}^{-1}$ respectively.
16. The spheres and stems were subtracted from the lung background activity fraction map.
17. The component mask values were replaced with activity concentration values, as measured in the radionuclide calibrator.
18. The component masks were summed to form a high-resolution ground truth activity image.

## Appendix 3: methodology for determining uncertainty on estimated kernel width

The uncertainty on the determined kernel FWHM for measured phantom data was estimated by computing the standard deviation of $h_{PVC}$ FWHMs derived by randomly sub-sampling half of the VOI voxels, see Table 3. 100 voxel sub-sampling realisations were conducted and the standard deviation calculated across all realisations.

This approach was validated using a 1-dimensional (1D) array of 700 elements of randomly assigned values between 0 and 1000. 1000 voxel sub-sampling realisations, randomly sampling half of the voxels, were conducted. $h_{PVC}$ was estimated for each realisation, minimising the RMSE over those voxels only, equation 4.

4 $$\begin{aligned} \textrm{RMSE} = \sqrt{\sum _v{(A_{\textrm{obs},v} - A_{\textrm{true},v})^2 / N}} \end{aligned}$$

where $A_{\textrm{obs},v}$ is the voxel value in the sub-sampled noisy observed array, $A_{\textrm{true},v}$ is the voxel value in the sub-sampled smoothed ground truth array, and *N* is the number of elements in the sub-sampled array, Fig. 11.

The standard deviation of the $h_{PVC}$ FWHMs from 1000 sub-sample realisations was calculated. In addition, 1000 noise realisations were generated and $h_{PVC}$ was computed (based on all voxels) for each realisation and the standard deviation of these estimates was computed. The ratio of the two standard deviations was used to determine the scaling factor that should be applied to the voxel sub-sampling uncertainty. The scaling factor was computed as 1.4 for a sub-sample factor of 2. As the standard deviation from the sub-sampling realisations was shown to be an overestimate of the uncertainty that would have been estimated from noise realisations, it is this uncertainty that is quoted in Tables 3 and 4.

## Abbreviations

PET: Positron emission tomography
CT: Computed tomography
IPF: Idiopathic pulmonary fibrosis
COPD: Chronic obstructive pulmonary disease
$$^{18}{}$$F-FDG: Fluorodeoxyglucose
AFC: Air fraction correction
AC: Attenuation correction
AF: Air fraction
FBP: Filtered back projection
MLEM: Maximum likelihood expectation maximisation
OSEM: Ordered subsets expectation maximisation
PSF: Point spread function
FOV: Field of view
FWHM: Full-width-half-maximum
XCAT: 4D eXtended CArdiac-Torso
GT: Ground truth
SUV: Standardised uptake value
TOF: Time of Flight
PF: Post-reconstruction filter
LAC: Linear attenuation coefficient
VOI: Volume of interest
HL: Healthy lung
RMSE: Root-mean-square-error
D710: GE discovery 710
UCLH: University College London Hospital
HRCT: High-resolution CT
VPFX: Vue point FX
EARL: EANM research GmbH
WMSE: Weighted-mean-square-error
MSE: Mean-square-error
NIfTI: Neuroimaging informatics technology initiative
Bq/ml: Becquerel per millilitre
DICOM: Digital imaging and communications in medicine
